# Are Prepubertal Gynaecomastia and Premature Thelarche Linked to Topical Lavender and Tea Tree Oil Use?

**DOI:** 10.17925/EE.2023.19.2.9

**Published:** 2023-08-31

**Authors:** Elsa W Braunstein, Glenn D Braunstein

**Affiliations:** 1. Department of Pathology, Cedars-Sinai Medical Center, Los Angeles, CA, USA; 2. Department of Medicine, Cedars-Sinai Medical Center, Los Angeles, CA, USA

**Keywords:** Gynaecomastia, premature thelarche, essential oils, lavender oil, tea tree oil, endocrine disrupting chemicals

## Abstract

Various studies, conducted since 2007, have reported a total of eight boys with prepubertal gynaecomastia and four girls with premature thelarche following exposure to lavender and/or tree tea oil. All patients experienced regression of the breast tissue after they stopped using these oils. Both of these essential oils, and several of their constituents, have oestrogenic and antiandrogenic activity *in vitro*. However, limited dermal penetration of some of the components means that the *in vitro* findings cannot be extrapolated to the *in vivo* situation. There are unanswered questions as to how much lavender or tea tree oil was actually present in the skincare products used by the children and a lack of information about exposure to other agents. Furthermore, since both prepubertal gynaecomastia and premature thelarche often spontaneously regress, it cannot be concluded that the use of lavender and/or tree tea oil is the cause of the gynaecomastia and thelarche in these children.

## Article highlights

- Lavender and tree tea oils have *in vitro* oestrogenic and antiandrogenic activity.
- It has been postulated that these oils may cause prepubertal gynaecomastia and premature thelarche.
- The association is based on a small number patients (N=12) described in five publications.
- Based upon the amount of dermal penetration of the oils or their constituents and the amount of these oils that would have to be applied for a long duration, it is unlikely that there is sufficient absorption to cause premature breast enlargement.
- Using Hill's criteria for evidence of causation, we conclude that the strength of association between these essential oils and premature breast development is weak, and that a cause-and-effect association has not been shown.

In 2007, Henley and colleagues proposed a link between the use of both lavender oil (LO) and tea tree oil (TTO) and the development of prepubertal gynaecomastia in three boys. They demonstrated that LO and TTO had oestrogen agonist and androgen antagonist activity *in vitro*, suggesting that LO and TTO were endocrine disrupting chemicals.^1^ Subsequently, Ramsey et al. extended this putative association to premature thelarche.^2^ Endocrine disrupting chemicals are natural or synthetic components that alter endocrine pathways by imitating, antagonizing or altering normal endocrine homeostasis.^3^ Gynaecomastia, which is usually caused by an imbalance between oestrogen-stimulating and androgen-inhibiting breast glandular tissue growth, is a common clinical condition associated with normal pubertal development, aging and various pathologies, including exposure to environmental endocrine disrupting agents.^4^ Prepubertal gynaecomastia is uncommon, usually idiopathic and in some instances has been linked to exogenous factors.^4,5^ Similarly, premature thelarche denotes early breast development in girls younger than 8 years, which also is idiopathic and transient in most instances.^6^ In this review, we will summarize the studies demonstrating the oestrogenic and antiandrogenic activities of LO and TTO, and critically analyze the reports of an association between use of these products and prepubertal gynecomastia and premature thelarche using Hill's criteria.^7^ We performed an Ovid literature search using search terms: “gynaecomastia”, “prepubertal gynaecomastia”, “premature thelarche”, “precocious puberty”, cross referenced with “lavender oil”, “tea tree oil”, “essential oils”, “endocrine disrupting agents”, “environmental estrogens” and “antiandrogens”. Additional articles were identified through the reference sections of retrieved articles.

## Lavender and tea tree essential oils

LO is derived by steam distillation from the flowers of the *Lavandula* genus of plants and has been used for centuries. Therapeutic benefits include antibacterial, antifungal, sedative and antidepressive properties, as well as smooth muscle-relaxing properties and healing effects for dermatitis, burns and insect bites.^8^ It is used in skincare products and shampoos because of its fragrance and its possible antimicrobial effects.^8^ LO contains a multitude of compounds, the main ones being linalool (20–45%), linalyl acetate (25–47%), 1,8-cineole (eucalyptol; <2.5%), β-ocimene, terpinen-4-ol (0.1–8%) and camphor (<1.2%).^7^ The two major components, linalool and linalyl acetate are absorbed through the skin, achieving peak plasma levels at 19 min.^9^

TTO is derived from steam distillation of the foliage and terminal branchlets of the *Melaleuca* genus and has been used in a variety of cosmetic skin, hair and nail products in concentrations of ≤2%.^10^ The main constituents are terpinen-4-ol (30–48%), α-terpinene (5–13%), γ-terpinene (10–28%), 1,8-cineole (eucalyptol, trace-15%), α-terpineol (1.5–8%), limonene (0.5–1.5%), terpinolene (1.5–5%) and p-cymene (0.5– 8%).^9^ Human skin penetration of each of the 15 tea tree oil components showed penetration of only terpinen-4-ol (3.7–8%) and α-terpineol (0.5– 1.1%) during the 24 h after application of a pure solution, and only 2.2–4% of terpinen-4-ol and no α-terpineol during the 24 h after application of a 20% TTO solution.^9^ Another study of human skin penetration confirmed the passage of terpinen-4-ol and α-terpineol through the skin, but also found eucalyptol could penetrate.^11^

The chemical composition of any essential oil varies depending on factors including the species of the plant, the soil conditions, climate, type of fertilizer, parts of the plants that are used, age of leaves and method of distillation.^12^

## *In vitro* and non-human *in vivo* studies of LO's and TTO's oestrogenic and antiandrogenic activity

In their initial report, Henley et al. performed an *in vitro* dose-response analysis of LO and TTO using the MCF-7 breast cancer cell line containing oestrogen receptors (ERα), to measure oestrogenic effects. The MCF-7 cells were transfected with an oestrogen-inducible luciferase reporter plasmid and three copies of an oestrogen receptor element (ERE), allowing the researchers to determine the response. Both LO and TTO stimulated an oestrogen-dependent response, with a maximum of 50% of the activity produced by 1 nM of 17ß-estradiol positive control. Tests with higher doses became cytotoxic to the cells. Additionally, LO and TTO increased the expression of the oestrogen-regulated genes, MYC, CTSD and IGFBP3. This response showed similar activity to the estradiol control (1 nM of 17ß-estradiol) and the response was reduced when treated with the ERα antagonist, fulvestrant. They also evaluated the possibility that the two essential oils exhibited antiandrogenic properties, using an androgen receptor (AR) assay in MDA-kb2 breast cancer cell line transformed with an androgen-inducible mouse mammary tumour virus-l uciferase reporter plasmid. Dihydrotestosterone (DHT) stimulates luciferase activity. When these cells were treated with the essential oils and DHT, suppression of luciferase activity occurred in a dose-dependent manner, like the effect of the antiandrogen flutamide, suggesting that components of the oils acted as an androgen-receptor blocker. They also showed that LO and TTO inhibited the expression of four genes stimulated by DHT: CYP4F8, Clorf116, UGT2B28 and SEC14L2. Henley and colleagues suggested that the oestrogenic and antiandrogenic effects of LO and TTO may be clinically important and “that susceptibility to gynaecomastia or other manifestations of endocrine disruption may require exposure to a threshold dose of these oils”. They further indicated that the threshold might depend on factors such as concentration of the LO or TTO in the product, the duration, frequency and quantity of the product that is used, and the genetic characteristics of the person exposed to the essential oils.^1^

Further studies from this group were reported by Ramsey et al.^2^ In addition to providing case reports on three girls with premature thelarche and one boy with prepubertal gynaecomastia – all of whom used LO, but not TTO – they provided extensive *in vitro* data supporting oestrogenic and antiandrogenic activity of individual components of the essential oils. Ramsey and associates began by testing the ERα ERE-mediated activity in cultured HepG2 cells transfected with human ERα and ERE and tested an estradiol control (E_2_), both LO and TTO, and several constituents of the products. E_2_ resulted in an almost 50-fold increase in the ERE-mediated promotor activity, while both essential oils showed an approximately 20-fold increase in activity. For the common constituents, α-terpineol showed the highest response (>8 fold increase), followed by terpinen-4-ol (<8 fold increase). Eucalyptol, dipentene/limonene and α-terpinene showed little to no activity response, while γ-terpinene showed a threefold increase at the highest dose. In the LO specific constituents, linalool showed a higher activity response (<8 fold increase) than linalyl acetate (fourfold increase). Using the same procedure with HepG2 cells cotransfected with SRC-2, a coactivator specific to humans, and ERα, E_2_, TTO, and LO showed significant increases in activity when SRC-2 was also present, while the essential oil components were more selective. Only terpinen-4-ol, α-terpineol, γ-terpinenel and linalyl acetate showed ERα activity in the SRC-2 cultured cells. A further examination, using HepG2 cells transfected with pG5 luciferase and pM-SRC-2-NR, showed that α-terpineol, linayl acetate and linalool can bind to the ligandbinding domain of ERα, and thus are able to activate SRC-2. To ensure the binding to the ERα led to a functional outcome, the gene expression of three ERα-regulated genes (GREB1, PGR, CTSD) was examined after treatment of the MCF-7 breast cancer cells with the essential oils and their constituents. Except for PGR expression in the presence of LO, all genes were expressed to a greater or lesser degree by the individual components, and the gene expression was inhibited by an ERα antagonist.

To evaluate the antiandrogenic properties that Henley et al. observed, Ramsey et al. used the promoter activity of the androgen receptor (AR) mouse mammary tumour virus (MMTV) in MDA-kb2 cells.^2^ If stimulated with androgens, the activity of MMTV will increase. When added along with testosterone, both LO and TTO significantly inhibited MMTV activity at all concentrations. Tetpinen-4-ol, α-terpineol, γ-terpinene, linalyl acetate and linalool reduced MMTV activity and no response or change in activity occurred when eucalyptol was present. The study also included an investigation of the essential oils’ effect on AR-regulated gene expression. Three AR target genes (SEC14L2, CYP4F8 and UGT2B28) were cultured with MDA-kb2 cells, and gene expression was evaluated with each oil and constituent. With all tested materials, SEC14L2 showed no gene expression. All constituents, except for eucalyptol and α-terpineol, blocked CYP4F8 activity. Only terpinen-4-ol, α-terpineol, linalyl acetate and linalool halted gene expression for UGT2B28.^2^ It should be noted that, while these results show oestrogenic and antiandrogenic properties for TTO, LO and their major constituents, the tests were done *in vitro* and cannot be assumed to verify an *in vivo* hypothesis.

Several groups affiliated with the essential oil industry have criticized the *in vitro* studies of Henley, et al. and Ramsey and co-workers on methodological grounds.^13–15^ They point to early studies demonstrating that polystyrene present in tissue culture dishes like those used by Henley and Ramsey contain oestrogenic substances such as alkylphenols, phthalates and bisphenol A, which can be released in the presence of essential oils and stimulate the MCF-7 cells. In contrast, vegetable oils like the ones used by Ramsey as a control to examine the possibility of such leaching does not do this to any appreciable effect.^16–18^ Additionally, the Henley group used Dulbecco's Modified Eagle Medium, which according to Larkman, enhances the leaching effect.^14^ However, Ramsey and colleagues did note that their experiments with essential oils showed varying activity and that some showed no activity. If the essential oils leached substantial amounts of oestrogenic substances out of the plastic, one would expect to see a positive oestrogenic effect in all the studies, which was not the case.^19^

Prior to the studies of Henley and Ramsey, Perry and colleagues examined the *in vitro* oestrogenic activity of the essential oil from a member of the lavender family, *Salvia lavandulaefolia* Vahl. (Spanish sage). Using the induction of β-galactosidase activity in a recombinant oestrogen-inducible yeast assay, they found the essential oil demonstrated weak oestrogenic activity, although it was not significantly different from the control because of high variability. However, geraniol, which comprised <1% of the essential oil, did show significant oestrogenic activity, while α-pinene, β-pinene, 1,8-cineole and thujone did not.^20^

Using a sensitive bioassay with recombinant yeast cells expressing the human ER, Howes and co-workers confirmed that geraniol had estrogenic activity, while eugenol, which is present in both LO and TTO, had antioestrogen effects.^21^ Both compounds were capable of displacing E_2_ from isolated ERs, but neither showed oestrogenic or anti-oestrogenic activity in an oestrogen-responsive human cell line (Ishikawa-Var I). In an androgen-inducible yeast screen, neither geraniol nor eugenol had androgenic or antiandrogenic activity.^21^

Nielsen used the MCF-7 BUS cell subline cell proliferation assay to compare the response of TTO and its constituents to the effect of graded doses of E_2_. TTO showed a weak, but dose-dependent oestrogenic response, with the maximum response being 34% of the peak E_2_ response. Neither terpinen-4-ol nor α-terpineol or eucalyptol induced cell growth above 10% of the maximum E_2_ response. Terpinen-4-ol slightly reduced the E_2_ stimulation in this assay, while α-terpineol caused a significant inhibition indicating an anti-oestrogenic activity. Of importance, none of the TTO constituents which have been shown to be absorbed through the skin alone or in combination showed oestrogenic activity. Based on these findings, the author concluded that *in vivo* results cannot be extrapolated from the *in vitro* responses and, if there is a connection between the application of TTO and endocrine disrupting activities, there must be another causation factor. That factor could be another constituent that penetrated the skin unnoticed, however, this is highly unlikely.^22^

Simoes and associates utilized an oestrogen response element-l uciferase reporter assay with MCF-7 cells to examine the oestrogenic and anti-oestrogenic activities of essential oils. In contrast to the studies by Henley and Ramsey, LO demonstrated non-significant minimal stimulation of the ERE-l uciferase activity and little or no stimulation of MCF-7 cell growth.^23^

An analysis of the oestrogenic and anti-oestrogenic activity of ethanol extracts of hair and skincare products utilizing a cell proliferation assay with the oestrogen sensitive MCF-7:WS8 cell line showed that the product containing TTO had no oestrogenic activity, but did exhibit anti-oestrogenic activity, while the product with geraniol showed neither oestrogenic nor anti-oestrogenic activity.^24^

A recent study, reported only in abstract form, indicated that some essential oil metabolites, including eucalyptol, inhibited CYP17A1 (17α-hydroxylase and 17,20-l yase) activity in an adrenal cell line.^25^ This could potentially decrease androgen production if the metabolites had sufficient skin penetration. The authors also indicated that some constituents inhibited CYP19A1 (aromatase) activity, which would potentially reduce oestrogen production.^25^ Indeed, in an *in vitro* assay using placental cells, eucalyptol and limonene decreased E_2_ production, while 4-terpineol increased progesterone and E_2_ secretion in a doseindependent manner.^26^ Again, as with the other studies summarized above, it is not possible to extrapolate the *in vitro* findings to the *in vivo* situation where absorption, distribution, metabolism and excretion exert dominant roles on tissue exposure and response.

The disparate results from the different *in vitro* studies likely relate to differences in the assay methodology, as well as the source, potential contaminants in the preparations, and concentrations of the multiple constituents in the essential oils.

In addition to the *in vitro* studies, there exist a small amount of animal data, which also have conflicting results. When tested *in vivo* using a uterotrophic assay in immature female rats exposed to LO through the dermal route in doses that were equivalent to 6,000–30,000 times the maximum daily human exposure, and greater than 5,000–1,000,000 times the estimated exposure from hair and skincare products that may be used by prepubertal boys, Politano and co-workers concluded that, at the dosages tested, LO is not an oestrogenic agent.^27^ Howes et al. examined the effect of geraniol in ovariectomized mice and did not find the usual oestrogen-induced uterine hypertrophy or acute increase in uterine vascular permeability.^21^ Exposure of prepubertal female rats to LO via nasal spray or indoor exposure via a LO diffuser triggered early puberty with earlier vaginal opening, significantly higher folliclestimulating hormone (FSH), luteinizing hormone (LH) and non-significantly higher E_2_ levels than in control rats. This is not what would have been expected with LO having *in vivo* oestrogenic activity, which would have suppressed gonadotropins and E_2_. Indeed, the authors thought that the mechanism of action was probably through olfactory stimulation of the hypothalamus, rather than a hormonal effect.^28^ In contrast to the above studies showing no *in vivo* oestrogenic effect of LO, Slighoua and co-workers reported an increase in uterine weights and serum E_2_ levels of female rats treated with hydro-ethanolic extracts of *Lavandula officinalis* Chaix.^29^ These extracts contained phenolic compounds, but it is unknown whether they also contained linalool or linalyl acetate, as found in LO. The results of the *in vitro* and *in vivo* studies are summarized in *Table 1*

There have been a few human studies using essential oils that have examined the effects on reproductive hormones. Cohn and associates studied topical application of LO and TTO in postmenopausal women with hot flashes. There were no significant differences between on treatment and baseline FSH levels and hot flash frequency or severity, and the investigators concluded that LO and TTO did not have oestrogenic effects.^30^ Heger-Mahn et al. performed a double-blind, randomized, placebo-controlled trial examining the effects of an oral LO preparation on the pharmacokinetics and pharmacodynamics of an ethinyl estradiol/levonorgestrel oral contraceptive in adult women, and did not demonstrate any relevant changes in the parameters or in the levels of progesterone, E_2_ or sex-hormone-binding globulin. There were no effects on mean endometrial thickness or ovarian follicle size.^31^ In a study of peri-and postmenopausal women exposed to aromatherapy with different essential oils, there was no effect of LO on salivary oestrogen concentrations.^32^ Thus, the human studies that have examined possible clinical oestrogenic activity of LO or TTO have not shown it to be present.

Investigating whether LO and TTO may have clinical antiandrogenic effects, Tirabassi et al. examined the effect of a LO and TTO spray on women with idiopathic hirsutism. This randomized, open-label, placebo-controlled study involved treating the hirsute areas twice daily for three months. There were no significant differences between groups or between baseline and three months in the levels of FSH, LH, E_2_, total or free testosterone, progesterone, 17-hydroxyprogesterone, sex-hormone-binding globulin, dehydroepiandrosterone sulfate, or Δ^4^-androstenedione.^33^ Nevertheless, there was a statistically significant decrease in the Ferriman-Gallway hirsutism score and hair diameter, suggesting a topical effect on hair growth rather than a systemic effect.^33^

**Table 1:** *In vitro* and *in vivo* biological activity of LO and TTO and their constituents

Substance	*In vitro* estrogenic activity (References)	*In vitro* antiandrogenic activity (References)	*In vivo* estrogenic activity (References)
LO		+ (1,2,11)	+ (1,2)	-rat uterotrophic assay ^(27)^
-(23)		+ rat uterotrophic assay ^(29)^
Linalyl acetate	+ (2)	+ (2)	ND
Linalool	+ (2)	+ (2)	ND
Geraniol	+ (20,21)	-(21)	-mouse uterotrophic assay ^(21)^
α and β-Pinene	-(12)	ND	ND
TTO	+ (1,2,22–24)		
α-Terpinene	-(2)	+ (2)	ND
γ-Terpinene	-(2)	+ (2)	ND
LO and TTO			
Eucalyptol		-(2,22)	+ (25)	ND
	-(2)	
4-Terpinenol	+ (2)	+ (2)	ND
Limonene	-(2)	+ (2)	ND
α-Terpineol		+ (2)	+ (2)		ND	
-(22)

*-= no activity shown; + = shows activity; ND = Not determined.*

## Reports of prepubertal gynaecomastia and premature thelarche in children exposed to LO and/or TTO

As noted above, the putative association arose from the study of Henley et al., who described three prepubertal males diagnosed with gynaecomastia, each of whom used regular topical applications of lavender and tea tree products.^1^ However, the product names or ingredients were not identified in the report. Each otherwise healthy boy had spontaneous breast development. Patient 1, aged 4 years and 5 months, had been exposed to a LO-infused “healing balm” before the initial diagnosis. After ceasing application of the balm for 4 months, his breast size decreased from 2.5 cm by 2.5 cm to 1.5 cm by 1.5 cm.^1^ A second patient (patient 2), aged 10 years and 1 month, used a hair gel and shampoo containing both LO and TTO every morning. Both he and his mother noticed that the condition was more pronounced in the evening than the morning. Within 9 months of ceasing to use the hair products, the patient's gynaecomastia had resolved. Patient 3, aged 7 years and 10 months, regularly used a lavender-scented soap and occasionally applied lavender-scented skin lotion. After he ceased using the products, his gynaecomastia resolved. His fraternal twin used the same lavender-scented lotion but did not exhibit gynecomastia.^1^ After showing *in vitro* oestrogenic and antiandrogenic activity of LO and TTO, the authors proposed that these essential oils were the cause of the prepubertal gynaecomastia.^1^

Along with the three initial patients described by Henley et al., an additional nine patients who were reported to use a product containing LO or TTO were subsequently diagnosed with either prepubertal gynaecomastia (total n=8) or premature thelarche (n=4) (*Table 2*).^1,2,27–29^ Three of the eight boys had Tanner genitalia staging, and all were stage 1. Eight of the twelve children also had Tanner pubic hair staging. Seven of the eight were also Tanner stage 1, while one was Tanner stage 2.1,2,34,35 The latter (patient 2 in *Table 2*) had an increase in free testosterone and DHEA-S, suggesting that he had transitioned to early puberty. Only two of the twelve patients used a product containing TTO. One (patient 2 in Henley et al.) used a styling gel and shampoo with both LO and TTO. The other patient, described by Lopez-Rodriguez and Marcos, used a shampoo, to which four drops of TTO was added to prevent head lice.^34^ This was the only patient described who used TTO exclusively. Diaz et al. reviewed three patients who presented with prepubertal gynaecomastia.^35^ Of the three, two used the product “Aguas de Violetas”, a lavender-based cologne. The other patient used an unidentified shampoo for a year. When each patient ceased application of the products, the gynaecomastia resolved within months (see *Table 2*). In the publication by Ramsey et al., four more patients were recorded with prepubertal breast development.^2^ One boy (patient 8), aged 7 years and 11 months, used “Crusellas Violet Water”, and was reported to have gynaecomastia for 4 years, before he discontinued his use of the lavender-infused product and it was resolved. One girl (patient 10), aged 7 years and 6 months, used “Mi tesoro Agua de Violetas” continuously until advised to stop, and her thelarche resolved 6 months later.^2^ Another patient (patient 11), reported to have premature thelarche for almost 3 years, had used “Baby Magic Calming Baby Bath Lavender and Chamomile” since infancy but, on stopping, her breast development resolved.^2^ The last patient (patient 12) recorded by Ramsey et al. sat next to her teacher's LO diffuser for 1 year at school. During that year, she was recorded to have premature thelarche, and upon switching seats, it resolved.^2^ One other patient (patient 9) reported to have premature thelarche used wipes, shampoo, body wash, moisturizing cream and skinsmoothing lotion containing LO. Her thelarche resolved after ceasing use of LO infused products.^36^ In each of the patients noted above, there was a clear temporal relationship between the use of LO and/or TTO and the appearance of premature breast development. Additionally, the breast tissue regressed or disappeared after discontinuation of the LO or TTO. None reported a rechallenge test with the product thought to cause the breast enlargement. This is especially important, since both prepubertal gynaecomastia and premature thelarche often regress spontaneously.^5,6^

Unlike the above reports with patient-specific details, Block reviewed the charts of about 1,100 children under six years in his practice and noted that 33 under the age of 5 had prepubertal gynaecomastia (n=5) or premature thelarche (n=28). Twelve of these 33 patients had a history of LO or TTO use. However, the relationship of this to the onset of the breast development, the name of the product, duration of use, and whether the breast enlargement resolved while they were using the LOor TTO-containing skin or scalp creams or lotions, or whether the breast enlargement disappeared after cessation of the use of the skin lotions, was not described.^37^

These reports have been heavily criticized on several grounds, with most of the criticism emanating from scientists associated with the essential oil industry raising the possibility of commercial bias.^13–15,38–43^ First, there has been considerable controversy as to whether the products used by some of these children actually contained LO or TTO. As noted, the skincare products and shampoos used by the children in the Henley et al. study were not named, but the authors indicated that they had found the ingredients listed on the manufacturers’ websites.^44^ A concern was raised that three of the products used by patients in the Ramsey et al. study may have been mislabelled as containing LO. Assays of the products “Crusellas Violet Water” cologne and “Baby Magic Calming Baby Bath Lavender and Chamomile” soap were done by gas chromatography-mass spectrometry and liquid chromatography with tandem mass spectrometry.^13^ LO was not found to be a component of the Crusellas Violet Water cologne, however, another potential endocrine disruptor, diethyl phthalate, was. The Baby Magic Calming Baby Bath soap had a composition containing a “very low” amount of LO.^13^ A further study was done on the products “Crusellas Violet Water” cologne and “Mi Tesoro Agua de Violetas” cologne, along with several other known products labeled “aguas” (Augustin Reyes, PMB Agua de Violetas, AFFA Violetas Francescas). The assay showed that the composition of all products did not contain linalyl acetate. Except for the Augustin Reyes product, which had a 2.28% composition of linalool, the products also did not contain linalool.^14^ The 2.28% composition of linalool in the Augustin Reyes product falls well below the ISO Standard of 25–38%.^39^ Conversely, Diaz et al. used high performance liquid chromatography and mass spectroscopy to analyze the “agua de violetas” cologne used by one of his patients (Patient 6 in *Table 2*) and identified both linalool and linalyl acetate confirming the presence of LO in the product.^35^

**Table 2:** Summary of reports of prepubertal gynecomastia and premature thelarche and LO and/or TTO use

First author, year	Patient	Age	Breasts	Duration of gynaecomastia	Testes	Genitalia Tanner stage	Pubic hair Tanner stage	Laboratory results	Product used	Outcome after discontinuation
**Prepubertal gynaecomastia (N=8)**										
Henley, 2007^1^	1	4 5/12	Bilateral 2x2 cm	2-3 weeks	3 mL	1	N/A	Nl TFT, LH, FSH, T E2, DS, 17OHP, biochemistry panel	Lavender healing balm	Resolved 4 months after discontinuation
		4 8/12	2.5x2.5 cm, tender	15-16 weeks						
	2	10 1/12	Bilateral 3.5x4x3.5 cm, tender	5 months	3 mL	1	2	Free T and DS slightly f	Styling gel with LO and TTO	9 months later 1 cm "without glandular tissue"
	3	7 10/12	Bilateral, "Tanner 2"	1 month	3 mL	1	N/A	Nl LH, FSH, E1, E2, E3, Total E, DS, 17OHP, TFT, hCG, biochemistry panel	Lavender-scented soap and skin lotion	Resolved after discontinuation
Lopez-Rodriguez, 2014^34^	4	9	Left breast 3 cm long, 2 cm high	1 month	3x3cm	N/A	1	Nl LH, FSH, E2,T, DS, Prl,TSH, FreeT4, bone age	Shampoo with TTO	Resolved 2 months after discontinuation
Diaz, 2016^35^	5	9 7/12	Right breast 2x2 cm	When seen at 9 7/12 years	2 mL	N/A	1	Nl LH, FSH, A, T, DS, E1, E2, bone age.	Unidentified shampoo for 1 year	Resolved 4 months after discontinuation
	6	9 9/12	Right breast 5x6x4 cm (Ultrasound at 9 4/12 years was 3x1 cm)	1 year	2 mL	N/A	1	Nl LH, FSH,A,T, Free T E1,E2, E3, hCG, DS, Prl, bone age	Aqua de Violetas daily since childhood	Resolved soon after discontinuation
	7	9 4/12	Right 4x4 cm; Left 2x2 cm	3 years	2 mL	N/A	1	Nl LH, FSH,A,T, Free T E1,E2, DS, hCG, Prl; bone age =11 years (<2 SD)	Agua de Violetas frequently since childhood	Resolved 4 months after discontinuation
Ramsey, 2019^3^	8	7 11/12	Bilateral 4x4x3 cm	4 years	1 mL	N/A	N/A	Nl LH, FSH,T, E1,E2, 17OHP, Prl, bone age, chromosomes,	Crusellas Violet Water Cologne since infancy	Resolved 6 months after discontinuation
**Premature thelarche (N=4)**										
**Author**	**Patient**	**Age**	**Breasts**	**Duration**	**Pubic hair Tanner stage**	**Laboratory results**	**Product used**	**Outcome after discontinuation**		
Linklater, 2015^36^	9	1 2/12	Bilateral breast Development 2.5 cm	8 months	N/A	Nl LH, FSH, E2, hCG, liver function tests, pelvic and liver ultrasound, brain MRI, bone age. Prl and AFP slightly increased but both returned to normal.	LO in wipes, shampoo, body wash, moisturizing cream, skin smoothing lotion	Receded after discontinuation		
		2 2/12	3 cm bilaterally	20 months			Same			
Ramsey, 2019^2^	10	7 6/12	Left breast bud 4x3 cm	18 months	1	Nl LH, E2, TFT, bone age	LO cologne Mi Tesoro Agua de Violetas since childhood	Resolved 6 months after discontinuation		
	11	3 11/12	Right breast 3x3 cm	2 11/12 years; tender x2 months	1	Nl bone age	Baby Magic Calming Baby Bath Lavender and Chamomile exposure since infancy	Resolved 6 months after discontinuation		
	12	7 9/12	Tanner 2 left breast tissue		1	Nl bone age; uterus enlarged to peripubertal size on pelvic ultrasound	Exposed to LO diffuser for 1 year at school	Regressed by 3 months after discontinuation		

*A = androstenedione; AFP = alpha fetoprotein; DS = dehydroandrostenedione sulfate; E_t_ = oestrone; E, = oestradiol; E_3_ = oestriol; ESH = follicle stimulating hormone; hCG = human chorionic gonadotropin; LH = luteinizing hormone; N/A = Not available; Nl = normal; 170HP = 17a-hydroxyprogesterone; Prl = prolactin; T = testosterone; T4 = thyroxine; TFT = thyroid function tests; Dotal E = total oestrogens.*

**Table 3:** Table 3: Application of Hill criteria for causation to exposure to LO and/or TTO and prepubertal breast development

Hill criteria	Lavender oil	Tea tree oil	Comments
Strength	Weak	Weak	LO and TTO products are used by millions of people, and only 12 patients have been described in detail with prepuberal breast development, which occurs in children for a variety of reasons.^5,6^
Consistency	Not demonstrated	Not demonstrated	A total of 12 patients in five reports uncontrolled for confounding factors does not fulfill this criterion. Hawkins et al. study, though flawed by potential biases, is not consistent with the anecdotal reports ^(43)^.
Specificity	Not demonstrated	Not demonstrated	
Temporality	Yes	Yes	As noted in Table 2, the 12 patients described in detail were noted to have breast enlargement after they had used LO and/ or TTO, and the enlargement improved or resolved after discontinuing the product.
Biological gradient	Yes and no	Yes and no	Henley et al. and Ramsey et al. clearly demonstrated an in vitro dose-response curve of estrogenic and antiandrogenic activity of LO, TTO, and some of their constituents (see Table 1)^1,2^. However, there have been no human data nor well-controlled animal studies to support a biological gradient.
Biological plausibility	Yes	Yes	The in vitro data from Henley et al. and Ramsey et. al. show that LO and TTO have oestrogenic and antiandrogenic activity, and thus are potentially capable of causing gynaecomastia if sufficient amounts of the endocrine-active agents are absorbed and can reach the breast glandular tissue in high enough quantities to stimulate growth.^1,2^ The dermal penetration studies indicate that the active ingredients LO are absorbed, while very little of those in TTO are, making the latter less biologically plausible as a cause.^9,11^
Coherence	Not demonstrated	Not demonstrated	Despite the increased sales of skincare and shampoo products containing LO and/or TTO, the incidence of prepubertal breast growth has remained constant.^43^
Experiment	No clear result	No clear result	None looking at association with breast stimulation in humans. No evidence of an oestrogenic effect in adults.^29–32^ Possible antiandrogenic effect on hair growth in hirsute women with topical application.^26^ Studies with rodents show conflicting effects.^23,26,28,30^
Analogy	Yes	Yes	There are several examples of premature breast development in children exposed to oestrogen-containing products.^4^

*LO = lavender oil; TTO = tea tree oil.*

There are several lines of evidence against TTO having any potential clinical effect regarding estrogen or antiandrogen activities. The second patient listed by Henley et al. was the only initial case reported using TTO, as well as LO.^1^ It has been reported that a further investigation into the products used by Patient 2 showed that there were very low concentrations of TTO in the shampoo and “virtually undetectable” amounts in the hair gel.^40^ The drops containing “*Melaleuca alternifolia* extract” added to the shampoo of a nine-year-old boy with gynecomastia (Patient 4 in *Table 2*) were not analyzed. However, as previously stated, although TTO does have estrogenic activity *in vitro*, the three penetrable constituents of TTO (terpinen-4-ol, α-terpineol, and eucalyptol) did not show any positive estrogenic activity when tested *in vitro* alone.^22^

Another criticism of the studies associating LO and/or TTO to prepubertal breast development relates to the extrapolation of the *in vitro* results to the *in vivo* situation. It was noted that in the Henley et al. studies, 600,000 to 1.4 million times more LO or TTO was needed to elicit the same effect as 1 nM of E_2_, which served as the positive control, and it was calculated that a 20 kg child would have to use about 40 bottles of TTO-containing shampoo for each application.^41,42^ In addition, because of the limited amount of time that some of the products, such as shampoos, have skin contact before being washed off, the limited transdermal passage of many of the compounds in the products which reduces bioavailability, the lack of *in vivo* estrogenic activity in several studies, the volatile nature of EOs, and the possibility of exposure to other endocrine-disrupting chemicals contaminating the preparations such as chlorpyrifos, has led many authors to question the association.15,21,23,26,27,40,42

The 12 patients summarized in *Table 2* represented uncontrolled small case series or anecdotal reports. There is an additional anecdotal report of an 8-year-old girl with a 6-month history of precocious puberty, who had used LO hair spray for 6 months before the onset of pubertal development, but there was no notation as to whether the pubertal manifestations regressed after discontinuing the hair spray.^45^ Hawkins and associates looked at the issue with an epidemiological approach. They initially noted that, after adjusting for a range of factors, the prevalence of prepubertal gynaecomastia had remained stable, while sales of TTO and LO have increased. One would expect to have seen a parallel increase in gynaecomastia and premature thelarche. This group conducted a cross-sectional study of children aged 2–15 years old in the United States recruited from an email database of the Franklin Health Research Center, social media and physicians’ offices. One of the inclusion criteria was that the patients (or their parents on their behalf) self-i dentified as users of natural personal care products. The participants filled out the Aromatic Plant Ingredients and Child Health Survey online. The primary outcomes were the self-report of a diagnosis of prepubertal gynaecomastia (the term included both gynaecomastia and premature thelarche) or precocious puberty, as well as delayed puberty, diabetes, thyroid conditions, Cushing syndrome, hypopituitarism or growth hormone deficiency. They recruited 556 children with a mean age of 6.33 years, of whom 86% were white and 54.8% were male. Almost three-quarters of the respondents had been exposed to LO and/ or TTO. In this group, nine patients had an endocrine-related disorder: precocious puberty (n=2), delayed puberty (n=1), hypothyroidism (n=4), and growth hormone deficiency (n=1). None had a diagnosis of prepubertal gynaecomastia. They stated that there was no difference in the prevalence of the nine endocrine disorders versus that expected in the general population. They concluded that there is no relationship with endocrine disruption and the use of LO or TTO.^43^ As acknowledged by the authors, this type of study has potential issues with both recall and selection bias.

## What is the strength of the putative association between prepubertal gynaecomastia and premature thelarche and the use of LO and/or TTO?

As emphasized by Hennekens and DeMets, “Causation is a judgement based on the totality of evidence that includes several positive criteria”.^46^ These criteria were described in a classic paper by Sir Austin Bradford Hill in 1965.^7^ The first, and most important, is the *strength of the association*. Is exposure to LO and/or TTO associated with a relatively large increase in prepubertal gynecomastia or premature thelarche after confounding factors are removed? Second is *consistency*; has the association been repeatedly observed by others in different settings and over time? Third is *specificity* between the exposure and the effect, realizing that the effect (e.g. prepubertal gynaecomastia) may have many causes. The fourth is temporality. When dealing with endocrine disruptive agents, the effect or disease must occur or worsen after the exposure and not before, realizing that the exposure may occur years before the condition becomes clinically manifest. Prepubertal gynaecomastia or thelarche should appear after exposure to LO or TTO. Also, for reversible conditions, such as gynaecomastia, the condition should improve or remit after the exposure to the inciting agent is removed. Hill's fifth criterion is the presence of a *biological gradient*, or dose-response curve. Does the incidence of the condition rise with increased exposure? The sixth is *plausibility*. In this case, is it biologically plausible that exposure to LO and TTO could cause premature breast development? The seventh criterion is *coherence*. Does the incidence of prepubertal breast glandular tissue development increase in parallel to the use of LO and/or TTO products by children? *Experiment* is Hill's eighth criterion. Ideally, such experimentation would be a randomized, double-blind, placebo-controlled trial of a large group of children exposed to either LO and/or TTO containing skincare products or shampoo or identical products without LO or TTO to compare the incidence of prepubertal gynecomastia or premature thelarche. Serial measurements of testosterone and E_2_, as well as serum levels of the LO and TTO metabolites, should be performed to see if clinically relevant concentrations of bioactive LO and TTO metabolites are reached and whether there are antiandrogenic effects or alterations in aromatase activity. Short of that, animal experimentation could lend support if the species chosen responds to the stimulus similar to humans. Hill's final criterion is *analogy*. Is there evidence that prepubertal gynecomastia or thelarche can occur from other environmental exposures? *Table 3* summarizes our scorecard for the association between LO and TTO and prepubertal gynecomastia or premature thelarche.

In summary, in applying the Hill criteria, we find that there is insufficient evidence to support a link between the use of LO or TTO products and the occurrence of prepubertal gynaecomastia or premature thelarche. We acknowledge that there is biological plausibility from the *in vitro* studies and that there is a temporal relationship between the use of these products and the onset of premature thelarche or prepubertal gynaecomastia in the 12 cases that have been reported in detail to date. However, the issues about the duration of exposure, the actual dermal absorption of the oestrogen agonist or androgen antagonist components of LO and TTO, the possibility of contamination of some of the products with oestrogenic substances, and the lack of unbiased, non-a necdotal human experimental results has led us to conclude that LO and TTO have not been shown to be associated with the clinical development of premature breast enlargement, and that the strength of association is very weak.
